# Supplementary material for: Mandatory membership of community-based mutual health insurance in Senegal: A national survey
Source: PLOS Glob Public Health. 2023 Sep 28;3(9):e0001859. doi: 10.1371/journal.pgph.0001859 (PMC10538694; doi:10.1371/journal.pgph.0001859)
Supplement: Attachment — Submitted filename: R1 d.docx [file pgph.0001859.s003.docx]

Dear Prof Osondu Ogbuoji

Academic Editor

PLOS Global Public Health

Thank you for your recommendation for revisions and your review. We are pleased to respond to each of the comments below, hoping that this will be sufficient for you to make a decision.

Sincerely

V Ridde for the authors

***

1. Please include the following request in the decision letter, and ping me with follow-up. “Please include a complete copy of PLOS’ questionnaire on inclusivity in global research in your revised manuscript. Our policy for research in this area aims to improve transparency in the reporting of research performed outside of researchers’ own country or community. The policy applies to researchers who have travelled to a different country to conduct research, research with Indigenous populations or their lands, and research on cultural artefacts. The questionnaire can also be requested at the journal’s discretion for any other submissions, even if these conditions are not met. Please find more information on the policy and a link to download a blank copy of the questionnaire here: https://journals.plos.org/globalpublichealth/s/best-practices-in-research-reporting. Please upload a completed version of your questionnaire as Supporting Information when you resubmit your manuscript.”

*** We completed the questionnaire. Please note that 3 of the authors (VR, IG, AF) live in Senegal, and the other two have carried out short missions to the country concerned by this study, where they have also been conducting research.

2. Please send a completed 'Competing Interests' statement, including any COIs declared by your co-authors. If you have no competing interests to declare, please state "The authors have declared that no competing interests exist".

***Done

3. Additional Editor Comments (if provided):

This is an important paper because of the potential contributions to UHC in Africa. It will need major revision before it can be accepted. Please submit a revision along with a completed STROBE checklist (Cross-sectional version found here: https://www.equator-network.org/reporting-guidelines/strobe/). Also address the comments from the reviewers below.

***STROBE checklist has been added

Your paper will also benefit from an English language editor.

*** The article was edited by an English-speaking editor, Heather Hickey

Reviewer #1: This is a very important study as African countries seek ways to promote UHC.

*** Thank you for this appreciation. We have tried to improve the article to better meet this need for knowledge gap.

However, there are a few observations and feedback you may consider in revising the manuscript.

In the introductory section, it may be useful to include the population of Senegal. It may also be useful to give information on the proportion of the population who own a mobile phone. This additional information will provide more context.

*** We have added details; thank you!

In line 126, the use of the words 'qualitative variable' may be misleading, and I suggest the use of 'categorical variable' as a replacement.

*** We have replaced it.

In the discussion section, starting at line 180, I will suggest summarizing your results before delving into comparisons with other studies.

*** We have added this section

In line 219 - 221, it is not quite clear to me what the state subsidies comprise of.

*** We have specified

Is it possible to have respondents who did not speak any of the major languages? is it possible to explain what was planned and what was done? It is also possible that some meanings may have been lost in the process of translation.

*** We have used the six main languages of the country, and we do not believe that it is possible that respondents do not speak any of these languages. In addition to French, the official language, the five other languages used are the national languages and represent 80% of the national languages spoken according to the latest population census (<https://id.erudit.org/iderudit/1054052ar>). We do not believe that this is possible because the questionnaires were administered by people who were fluent in one of the mother tongues but also had a good knowledge of the other two most widely spoken languages in Senegal, Wolof and French. The questions were asked in the primary language, but discussions could also be held with the respondents in case of difficulties in understanding. After discussion with the interviewers during the daily debriefing and the final debriefing of the survey, this translation issue was not raised.

concerning the figures, it may be necessary to provide a legend and appropriate axis labels. the figure and tables may also be better presented with brief explanatory footnotes.

*** We have added these details

Have consideration been given to the use of regression models for data analysis? This may help identify associations of some predictor variable with measured outcomes and possible confounding.

*** It was a telephone survey designed to obtain a national sample with very short notice (and at a lower cost). We did not have the means to carry out the study in households across the country, which would have allowed us to have more detailed data, especially on household characteristics. However, as you requested, we have added a regression analysis to analyse the relationship between trust and membership. A paragraph and a table have been added. We believe that this study can be considered the first on the subject in Senegal, and the primary purpose is to present descriptive and exploratory results that are useful for policymakers but also for researchers to show the relevance of a more detailed national study, but which will necessarily be much more expensive. We have added this limitation and reflection in the article, the last section of the Discussion.

Reviewer #2: The paper does not elaborate on objectives and specific hypotheses for testing through analysis of the quantitative survey dataset.

*** We have specified the article's objective. As this is exploratory research, the first on the subject in Senegal, we found it difficult to test a hypotheses. However, the discussion allows us to reflect on the issues surrounding the results. We have added the section "Design and Population under study" to provide details on this subject.

There is no information on what is the current levels of health insurance coverage in the country for both formal and informal workers/households.

*** In the introduction, we explained that "less than 5% of the population is affiliated with a CBHI" since the article is concerned only with CBHIs. However, we have added details on this subject in the "Setting" section with an additional reference, thank you

Statistical analysis is very rudimentary.

*** Unfortunately, for this exploratory study, which was carried out by telephone to obtain data on a national scale on very short notice (and at a lower cost), we do not have enough data for more complex statistical analyses. It would have taken too large a budget to carry out a nationally representative household survey that would give reliable data on household characteristics. However, as you requested, we have added a Tobit regression to analyse the relationship between trust and membership. A paragraph and a table have been added. These analyses are helpful in helping Senegalese decision-makers to reflect on the issues surrounding insurance contributions. This is now a very important political and technical issue in Senegal, as it is elsewhere in Africa, according to reviewer 1, and we hope that this initial research will lead to more funding for more in-depth studies and statistical analysis. We have added news sentences detailing this limitation and the need for further research to the article.

How the well-being is measured is not explained.

*** The welfare quintile is constructed in three stages:

First, all household goods for which information has been collected are assigned a weighting generated after principal component analysis:

Next, the scores are summed per household and standardized concerning a standard normal distribution with a mean of 0 and a standard deviation of 1;

Finally, households are ranked in ascending order, and the sample is divided into quintiles, i.e. five groups with the same number of people. The 20% of the population with the lowest total wealth scores become the individuals in the lowest quintile; the next 20% are considered the second wealth quintile, and so on.

We add this information to the paper.

On line 87-88 the average age is 19 years (of whom, General population, participants) which is not correct.

*** We have corrected it, thank you

Discussion need better articulation.

*** We have also revised the discussion and added on section
